# Effect of Action Verbs on the Performance of a Complex Movement

**DOI:** 10.1371/journal.pone.0068687

**Published:** 2013-07-03

**Authors:** Tahar Rabahi, Patrick Fargier, Ahmad Rifai Sarraj, Cyril Clouzeau, Raphael Massarelli

**Affiliations:** 1 Centre de Recherche et d’Innovation sur le Sport (CRIS, EA 647), Université de Lyon, Université Claude Bernard Lyon 1, Villeurbanne, France; 2 Département de Physiothérapie, Faculté de Santé Publique-I, Université Libanaise Rafic Hariri, Hadath-Beyrouth, Lebanon; 3 Unité de Recherche, Institut Supérieur d’Ostéopathie (ISOSTEO), Limonest-Lyon, France; French National Centre for Scientific Research, France

## Abstract

The interaction between language and motor action has been approached by studying the effect of action verbs, kinaesthetic imagery and mental subtraction upon the performance of a complex movement, the squat vertical jump (SVJ). The time of flight gave the value of the height of the SVJ and was measured with an Optojump® and a Myotest® apparatuses. The results obtained by the effects of the cognitive stimuli showed a statistically significant improvement of the SVJ performance after either loudly or silently pronouncing, hearing or reading the verb *saute* (*jump* in French language). Action verbs specific for other motor actions (*pince* = *pinch*, *lèche* = *lick*) or non-specific (*bouge* = *move*) showed no or little effect. A meaningless verb for the French subjects (*tiáo* = *jump* in Chinese) showed no effect as did *rêve* (*dream*), *tombe* (*fall)* and *stop*. The verb *gagne* (*win*) improved significantly the SVJ height, as did its antonym *perds* (*lose*) suggesting a possible influence of affects in the subjects’ performance. The effect of the specific action verb *jump* upon the heights of SVJ was similar to that obtained after kinaesthetic imagery and after mental subtraction of two digits numbers from three digits ones; possibly, in the latter, because of the intervention of language in calculus. It appears that the effects of the specific action verb *jump* did seem effective but not totally exclusive for the enhancement of the SVJ performance. The results imply an interaction among language and motor brain areas in the performance of a complex movement resulting in a clear specificity of the corresponding action verb. The effect upon performance may probably be influenced by the subjects’ intention, increased attention and emotion produced by cognitive stimuli among which action verbs.

## Introduction

The relation between language and motor action has been the domain of philosophy much before becoming that of neuroscience (see: [1]), but in the last decades numerous findings (e.g.: [2]–[6]) have reported the existence of a probable interaction among the areas involved in language and movement. This has been shown, for example, by the fMRI experiments of Hauk et al [3] showing that silent reading of action verbs *lick*, *pick* and *kick* might apparently activate somatotopically the cerebral motor areas corresponding, respectively, to tongue, hand and foot. Other results ([7], [8] and for review see: [9]) have supported the hypothesis that reading action verbs may create specific links between the cortical areas specialised in language, audition and motor action. For instance, during lexical decisions on words related to action, Pulvermüller et al [10] reported that these cortical areas are interconnected in a category-specific manner. In other studies it was found that reading action verbs related to the hand and the arm, facilitates the execution of a reaching movement, when compared to reading other verbs describing mouth and leg actions [11], [12]. Hearing sentences that describe hand and foot action may also modulate the corresponding effectors, as it has been verified directly by monitoring motor-evoked potentials [5]. In an fMRI experiment, listening to sentences describing actions performed with the mouth, the hand, or the leg activated a left-hemispheric fronto-parieto-temporal network, known to be activated by action execution and observation [6]. In line with these findings, Dalla Volta et al [13] found that opening the fingers of the hand in response to hearing hand-related verbs was faster than when foot-related verbs were heard; a similar effect was observed on arm velocity during reaching-grasping movements. Other experiments have confirmed that a link between action words and motor performance might indeed exist [14]–[17], probably through the mediation of a mental simulation process (for review see: [18], [19], *vide infra*). Some authors are however more cautious concerning a somatotopic, modal relationship between action verbs understanding and activation of motor areas (see: [20]–[23]), preferring instead a more amodal ‘grounded hypothesis’ of interaction among the concerned areas (see for review: [24], [25]).

Several hypotheses have been advanced to explain the relation of language and motor actions (see for review: [26], [27]). Some authors have suggested that gestures, in particular, may assume the role of a “primitive meaning processor” ([27], p 944) (see also: [28]), while others have advanced the possibility that the effect of words or phrases on the motor system might arise through the mental representation of a given motor action [18], [24], [29]. Mental representations may create mental images and the use of these has been well studied in the past under the term of *simulation* [30], [31], which was intended as the ‘mental rehearsal’ of a motor action (for reviews see: [32], [33]). Such procedure had been used by athletes and coaches in sport activities as early as the 1930’s [34], [35] and it has been renamed *kinaesthetic* imagery (KI) later on to clearly distinguish it from visual imagery. The latter represents the mental vision of a movement, while the former concerns the kinaesthetic sensation of the action in the absence of an actual execution [36]. The effects of KI on the amelioration of a gesture performance when executed before the actual motor action have been well documented for simple and complex motor action (for reviews see: [37], [38]). The overall research led on the subject suggests then that language, motor, audition, vision and associative areas, including Wernicke’s, form a peri-Sylvian circuitry [39] of sensory and motor neurons some of which (mirror neurons) may fulfil both functions (see for review: [9], [27], [40], [41]). This ensemble of cortical areas is partly involved also during KI (for review see: [29]).

The activation of brain functional areas, recruited when using various cognitive stimuli and found in most of the above cited studies, let suppose that the effect of language on the activation of motor areas (in particular premotor areas) may also lead to an improvement of the physical performance of an individual, i.e. having an effect upon the efficiency of a complex movement. Consequently, the experiments that will be presented were realised with the aim to observe the potential effects of action verbs upon the execution of a complex motor action commonly used in biomechanical studies, the Squat Vertical Jump (SVJ) [42]. The leading concept, in the present research, was to observe whether the reported activations solicited in the motor areas by action verbs might produce a macro-scale effect upon an action as complex as the SVJ or might instead resemble to the effect produced in simpler movements as those concerning fingers, hand or arm [11]–[13], [43]. The rationale was based upon results [44], [45] showing discordance in motor acquisition when actions of different complexity are executed. Wulf and Shea [44], pointing out that a ‘complex’ motor action has several degrees of freedom compared to a simpler one, have reached the expected conclusion that motor learning of simple movements is not the same as that of more complex ones, as in the case of the SVJ.

## Materials and Methods

### General Experimental Procedure

Action verbs were pronounced, heard or read at the second person of the imperative tense, in French, and the effects upon SVJ were studied in seven separate experiments. The possible influence of a specific action verb (*saute = jump*) upon the height performance of a SVJ was compared to the effect of KI, which was used here as a control for *jump* considering its known potentiality in improving complex motor action [46]. Possible non-specific effects were verified by: a. Mental calculus (subtractions of two digits numbers from three digits ones); b. verbs specifically describing motor actions other than jumping (*lèche* = *lick* and *pince = pinch*) or signifying a failing action (*tombe = fall*); c. verbs implying the involvement of affects (not specifying a particular movement but raising feelings and emotions in relation to the goal of the jump: *gagne = win* and *perds = lose*, or unrelated to the goal: *bouge = move* and *rêve = dream*, or contradicting the action: *stop*); d. Pronunciation and hearing of a Chinese verb (*tiáo* = *jump*) meaningless for French speaking subjects.

### Subjects

This study was approved by the Institutional Review Board of Claude Bernard University. Male French speaking subjects (a total of 114 students of the Faculty of Sport Sciences, University Claude Bernard Lyon 1, of the Institute of Osteopathy, ISOSTEO, Limonest-Lyon and of the Department of Physical Sciences of the Lebanese University, Beyrouth, Lebanon) were asked to participate to the experimental protocol and gave their written and informed consent. The anthropometric average characteristics of all the 114 subjects corresponded to the values for Caucasians (i.e. 104 Europeans: 23.02±2.8 years of age, 177.4±5.7 cm height and 72.3±6.4 kg weight and 10 Mediterraneans: 21.1±1.3 years, 172.9±2.8 cm height and 76.8±3.9 weight [47], [48]). The characteristics of the subjects in each experiment are shown in Table 1 and seven different groups of subjects were formed to participate to the seven experiments (one per each experiment). The subjects were briefly instructed before the execution of the experimental protocol, but they were left unaware of the expected results or of possible effects of a given action verb or of any stimulus used in the experimental protocol. The subjects performed SVJs without any previous particular expertise or training and, in this respect, they were considered naïve.

**Table 1:** Anthropometric characteristics of experimental subjects.

Experiment	1	2	3	4	5	6	7
Subject (n)	10	28	14	16	16	20	10
Age (years)	21.9±2.6	21.5±2.4	23.2±2.2	23.6±2.4	23.4±2.3	24.9±3.3	21.1±1.3
Height (cm)	176.2±4.1	175.7±6.9	179.2±6.6	178.4±5.2	177.0±5.3	178.7±4.3	172.9±2.8
Weight (kg)	72.1±4.5	70.3±7.0	75.0±7.4	73.4±5.6	70.9±6.7	73.2±5.8	76.8±3.9

As mentioned in the Material and Methods section, the 114 male subjects were from the Faculty of Sport Sciences (UFR STAPS, experiment 2 and 4), the Institute for Osteopathy ISOSTEO (experiments 1, 3, 5 and 6) and the Department of Physical Education of the Lebanese University (experiment 7). The presented values correspond to European anthropometric standards in the age range [47], [48]. No statistical difference among the anthropometric values was found (p<0.063, F = 2.0714).

### Jumps

The jumps consisted in classical maximal SVJ, i.e.: parallel feet, heels on the ground, separated as wide as the shoulders, knees bent at 90°, hands on hips during the whole jump executed without any noticeable countermovement [42], [49].

### Experimental Protocols

**Figure 1:**
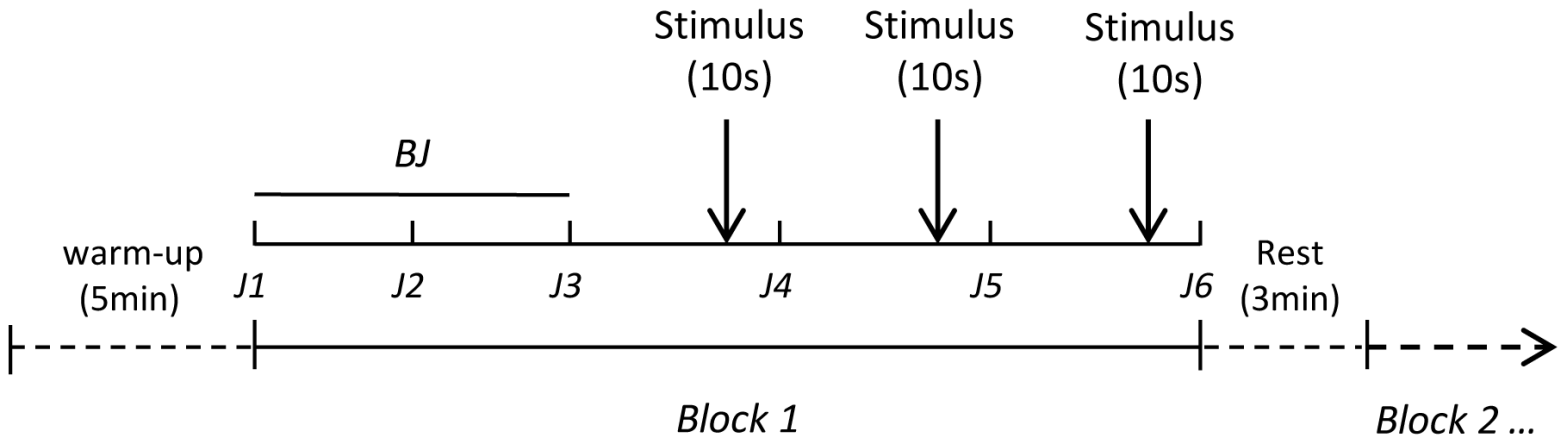
Protocol design. Before the start of the experiment the subjects were asked to perform some warm-up jumps, for about 5 minutes, to obtain a correct execution of the movement. The jumps consisted in classical maximal squat vertical jumps (SVJ). The heights of the jumps were measured as described in Material and Methods (section *Jump*). In a typical block the first 3 jumps were carried without cognitive conditions (to be called baseline jumps, BJ). The following 3 jumps were executed separately after the cognitive stimulus (repeated three times for 10 s before each jump). A rest period of 3 min was observed before a following block.

Before the start of each experiment the subjects were asked to perform some warm-up SVJs for about 5 minutes, to obtain a correct execution of the movement. Afterwards the participants were asked to perform a series of 6 SVJs (each series being called block) with a 3 min rest between blocks (see Figure 1). In a typical block the first 3 jumps were carried without any cognitive conditions (they were defined Baseline Jumps, BJ). The ensuing 3 jumps were executed after the cognitive conditions (lasting 10 s each; Figure 1) as specified below for each experiment. The blocks, comprising BJ and cognitive stimuli, were randomly presented to each subject.

Note that all subjects were asked to draw a particular attention to reach, during the warm-up period, the execution of correct SVJs and the maximal height in order to avoid improper variations in the height of the motor performance in the experimental follow-up. Moreover each subject was told not to communicate with other subjects about the experimental protocol.

### Apparatus and Measurement

The heights of the jumps were calculated, by measuring the time of flight, either by an OPTOJUMP® apparatus (Microgate France, 38330 St-Ismier; the apparatus was connected to a Laptop), in experiments 2 and 4, or by an accelerometer Myotest® Pro (Myotest France SAS, 84 210 Pernes les Fontaines), which was used for all other experiments.

A comparison experiment, performed on 8 subjects and using simultaneously both instruments, showed that the absolute heights measured with Myotest® might be higher (6.6 cm in the average) compared to OPTOJUMP®, a value that corresponds well to those found in the literature (Casartelli et al [50] reported values ranging from 5.7 to 6.9 cm). Insignificant differences were instead found between the two instruments in the measures of the effect of the action verb *jump* upon the height of the SVJ when compared, per cent wise, to BJ (when normalised to BJ, Optojump: 106.3±6.9% of height increase after pronouncing *jump*, n = 44, experiments 2 and 4; Myotest: 105.9±6.7%, n = 60, experiments 3, 5, 6 and 7 group effect: p = 0.76, t = - 0.30; the statistics was performed as specified below).

### Experiment 1: Reference Subjects

A control experiment was performed on 10 reference subjects (see Table 1) to check the presence of a possible effect of training. This was done according to the same protocol described in Figure 1 with the exception that no cognitive task was demanded to the subjects.

### Experiment 2: Pronouncing the Specific Action Verb and Comparison to Non-specific Stimuli

Twenty eight subjects (see Table 1) executed the following cognitive conditions: a. loud pronunciation of the specific action verb *jump* (the verb was repeated 9–10 times, during 10 s, with a steady, monotone, low voice), b. silent pronunciation (S-*jump*) was repeated in a similar way as when loudly pronounced, c. mental subtraction (MS) that was used as a control measure of a non-specific stimulus for the *jump* effect (the subjects were asked to mentally subtract two digit numbers from three digit ones) and d. kinaesthetic imagery (KI; the subjects were asked to feel the movement, without realising it) also used as a possible control for the effect of the action verb *jump*. Prior to the start of the experiment the subjects completed the revised version of the Movement Imagery Questionnaire (MIQ, [51]) to measure the individual ability in kinaesthetic imagery. No significant correlation was found between the values of MIQ and the performance after KI (r^2^ = **−**0.09; Pearson’s test).

### Experiment 3: Reading the Specific Action Verb

The action verb *jump* was projected on a black screen (the image was 1.3 m long, in diagonal) and the participants (see Table 1) stood at a distance of 3.5 m from the wall (as specified by the manufacturer, Optoma/ThemeScene® projector, 92100 Boulogne-Billancourt, France). The action verb was written in white (Police: Times New Roman, font 110) on a black background. The subjects carried out 6 cognitive conditions, each lasting 10 s: a. reading loudly *jump* (R-*jump*) written on the middle of the screen; b. reading silently the action verb (RS-*jump*); c. reading loudly *jump* that was moving bottom-up on the screen (Rm-*jump*; the movement speed of the verb was set on medium mode, Microsoft PowerPoint 2007® software); d. reading silently *jump* as in c (RSm-*jump*); e. pronouncing *jump* without visual support as in experiment 2, used as a first specific control; f. looking at the blank unlighted screen as a second control condition.

### Experiment 4: Pronouncing Non-specific Action Verbs

Sixteen participants (see Table 1) were asked to perform experimental blocks where the selected verbs either described the specific action (*jump* was used as the standard specific action verb) or actions concerning the fingers (*pinch*) or the tongue (*lick*), while a meaningless verb for all subjects (*tiáo* = *jump*, in Pinjing Chinese) was used as a semantic control.

### Experiment 5: Hearing Non-specific Action Verbs

The same experimental protocol used in the previous experiment was here performed with the difference that verbs were not pronounced but were heard by 16 subjects (see Table 1). The action verbs were previously recorded by an experimenter and pronounced once per second with the same tone and pace employed when the subjects pronounced them. The recording was listened for 10s before the execution of the SVJ.

### Experiment 6: Pronouncing Emotion and Feeling Verbs

Twenty subjects (see Table 1) took part in this experiment and five verbs were selected as cognitive stimuli: *gagne* (*win*), *perds* (*lose*, considered as the antonym of the previous), *rêve* (*dream*), *bouge* (*move*) as a general action verb and *jump* as the control specific action verb.

### Experiment 7: Hearing Actions Verbs Contradicting SVJs

Ten francophone subjects (see table 1) heard the verbs *saute* (*jump*), *tombe* (*fall*) and *stop* before the execution of the SVJ. The verbs were heard and not pronounced in order to produce a surprise in the subjects; the verbs were thus registered and listened by the subjects as in experiment 5.

### Statistical Analysis

Statistical analysis was performed using the software R2.11.1® (www.r-project.org). Analysis of variance for repeated measures (rANOVA) was used to examine the effect of the various conditions followed by the post-hoc Tukey’s test. Both were combined with the linear Mixed Effects Model [52], which was used to compare different conditions and taking into consideration the inter subjects’ variability. Statistical significance was set at 2-tail p<0.05.

## Results

### Experiment 1

This experiment was performed to show whether the repetition of SVJ might have a training effect upon the performance of reference subjects. The results (Table 2; group effect: p = 0.55, F = 0.76) showed that this was not the case. Moreover when the data were normalized to BJ (in per cent of BJ height) they showed a non-significant decrease in the height values (from 96.4±5.7% for J1 to 99.0±8.9% for J4; Table 3).

**Table 2:** Effect of action and other verbs upon the height of the SVJ.

Experiment 1 (n = 10)							
Condition	BI	J1	J2	J3	J4	–	–
mean±SD	31.8±4.3	30.6±3.5	31.1±3.9	31.1±3.7	31.4±3.2	–	–
p–value	–	.52	.95	.95	.99	–	–
z–value	–	1.95	1.14	1.13	–.88	–	–
**Experiment 2 (n = 28)**							
Condition	BJ	KI	Jump	S–Jump	MS	–	–
mean±SD	29.2±3.8	30.6±4.4	31.0±4.2	31.0±4.9	30.4±4.1	–	–
p–value	–	<.01	<.001	<.001	.02	–	–
z–value	–	3.49	4.66	4.68	3.13	–	–
**Experiment 3 (n = 14)**							
Condition	BJ	Jump	R-Jump	RS-Jump	Rm-Jump	RSm-Jump	Blank screen
mean±SD	32.6±3.6	35.3±2.9	34.8±3.0	34.8±2.9	35.1±2.6	34.7±2.9	32.6±3.1
p-value	–	<.001	<.01	<.01	<.001	<.01	1
z-value	–	−5.00	−3.97	4.04	−4.55	3.80	.05
**Experiment 4 (n = 16)**							
Condition	BJ	Jump	Lick	Pinch	Tiào	–	–
mean±SD	29±5.5	30.6±5.2	29.5±4.9	29.9±4.7	29.2±5.0	–	–
p-value	–	<.01	.78	.13	.99	–	–
z-value	–	3.72	1.14	2.34	.84	–	–
**Experiment 5 (n = 16)**							
Condition	BJ	H-Jump	H-Lick	H-Pinch	H-Tiào	–	–
mean±SD	32.7±4.0	34.5±3.9	33.5±3.9	33.2±4.5	32.8±4.9	–	–
p-value	–	<.001	.30	.83	.99	–	–
z-value	–	4.13	1.94	1.04	.21	–	–
**Experiment 6 (n = 20)**							
Condition	BJ	Jump	Win	Lose	Move	Dream	–
mean±SD	33.4±3.6	34.8±3.5	35.0±3.6	34.7±4.3	34.2±3.4	34.0±3.9	–
p-value	–	.006	<.001	.018	.36	.64	–
z-value	–	3.49	−4.10	3.19	−1.96	1.54	–
**Experiment 7 (n = 10)**							
Condition	BJ	H-Jump	H-Fall	H-Stop	–	–	–
mean±SD	29.8±0.9	31.7±0.4	29.8±0.7	29.9±0.7	–	–	–
p-value	–	<.001	.99	1	–	–	–
z-value	–	7.97	−.13	.10	–	–	–

Squat Vertical Jumps (J_1_ to J_4_, SVJ) were executed, in experiment 1, in the absence of cognitive stimuli; BJ: control jump; experiment 2 was performed to observe the effect of various cognitive stimuli such as KI (kinaesthetic imagery), the pronunciation of the specific action verb *jump* or its silent pronunciation (S-*jump*), subjects also performed a mental subtraction (MS, three digits minus two digits, the result was told at the end of the experiment); experiment 3 studied the reading (R) of *jump* under different modalities: the control was its pronunciation as in the precedent experiments, *R*: the subjects were asked to read loudly or silently (RS) the verb written on a screen, in Rm the verb was read loudly while moving bottom-up on the screen (moderate speed on Power Point software), (RSm) idem as in Rm, but the reading was silently performed, as control (*blank screen*) the subjects were asked to jump in front of the white (not lighted) screen; the effect of other action verbs (*lick, pinch* and *tiáo*) was studied in experiment 4; hearing action verbs (H) was studied in experiment 5 where subjects heard the same action verbs that in experiment 4 (H-*lick, H-pinch* and H-*tiáo*), through the voice of an experimenter, and in experiment 7 (H-*jump*, H-*fall* and H-*stop*). In experiment 6, the effect of the pronunciation of other non specific verbs upon the SJV height was realised with verbs *jump* (used as control), *win*, *lose*, *move* and *dream*. In all experiments the cognitive stimuli were randomized. The data are expressed as cm ± standard deviation (SD) and probability p and the z-score.

**Table 3:** Normalized Data: Influence of different cognitive stimuli upon SVJ performance.

Experiment 1 (n = 10)							
Condition	BI	J1	J2	J3	J4	–	–
Mean±SD	100.0%	96.4±5.7	98.1±8.5	98.2±8.6	99.0±8.9	–	–
p-value	–	.45	.90	.92	.99	–	–
z-value	–	1.67	.98	.83	.44	–	–
**Experiment 2 (n = 28)**							
Condition	BJ	Jump	S-Jump	KI	MS	–	–
Mean ±SD	100.0%	106.5±7.5	106.1±7.7	104.6±5.8	104.4±7.8	–	–
p-value	–	<.001	<.001	.008	.014	–	–
z-value	–	4.69	4.40	3.31	3.16	–	–
**Experiment 3 (n = 14)**							
Condition	BJ	Jump	R-Jump	RS-Jump	Rm-Jump	RSm-Jump	Blank screen
Mean ±SD	100.0%	108.9±11.0	107.0±7.3	107.3±9.8	108.2±8.3	106.9±8.9	100.1±4.7
p-value	–	<.001	.001	.001	<.001	.003	1
z-value	–	−5.00	−3.97	4.04	−4.55	3.80	.05
**Experiment 4 (n = 16)**							
Condition	BJ	Jump	Lick	Pinch	Tiào	–	–
Mean ±SD	100.0%	106.1±5.8	102.3±5.9	104.4±8.7	101.4±7.2	–	–
p-value	–	.001	.61	.043	.91	–	–
z-value	–	3.79	1.42	2.78	.86	–	–
**Experiment 5 (n = 16)**							
Condition	BJ	H-Jump	H-Lick	H-Pinch	H-Tiào	–	–
Mean ±SD	100.0%	105.5±5.3	102.6±3.6	101.2±4.4	100.1±5.3	–	–
p-value	–	<.001	.30	.89	1	–	–
z-value	–	4.13	1.92	.91	.05	–	–
**Experiment 6 (n = 20)**							
Condition	BJ	Jump	Win	Lose	Move	Dream	–
Mean ±SD	100.0%	104.1±4.0	104.9±5.8	103.6±5.5	102.5±5.9	101.9±6.1	–
p-value	–	.003	<.001	.014	.22	.55	–
z-value	–	3.70	−4.37	3.25	−2.24	1.67	–
**Experiment 7 (n = 10)**							
Condition	BJ	H-Jump	H-Fall	H-Stop	–	–	–
Mean ±SD	100.0%	106±3.7	99.7±2.1	100.1±2.5	–	–	–
p-value	–	<.001	.98	.99	–	–	–
z-value	–	7.35	−.39	.12	–	–	–

The absolute results in centimetres (see table 2) were normalized with respect to the respective baseline jumps (BJ) to give the per cent values of increase or decrease produced by the various stimuli.

### Experiment 2

The experiment showed a significant group effect (p<0.001, F = 7.32) and an improvement in SVJs was observed when the subjects pronounced loudly the verb j*ump* (p<0.001, z = 4.66; Table 2) or when they did so silently (without moving lips or tongue) prior to the jump (S-*jump*; p<0.001, z = 4.68). Kinaesthetic Imagery produced an increase in height (p<0.01, z = 3.49), thus confirming the potentiality of KI upon the motor execution of even a complex motor task as the SVJ. A height improvement was also obtained when the performed cognitive stimulus was a mental subtraction (MS; p = 0.02, z = 3.13). The effect of each cognitive condition revealed that the height of SVJ was significantly improved also when the data were normalized to BJ (Table 3) (from 104.4±7.8% for MS, p = .014, z = 3.16 up to 106.5±7.5%, p<.001, z = 4.69 for *jump*).

### Experiment 3

The results of the third experiment (Table 2) revealed the effect of adding a visual stimulus to the loud or silent pronunciation of *jump*. Reading (R) the action verb shown on a screen was effective in increasing the performance of SVJs either if read and loudly pronounced (R-*jump*; p<0.01, z = −3.97) or if silently read (RS-*jump*; p<0.01, z = 4.04), but it was not significantly different from the loud pronunciation of the verb without reading (*jump*; p<0.001, z = −5.0; Table 2). When the action verb was moving bottom-up on the screen, no additional effect was observed (Rm-*jump*: p<0.001, z = −4.55; RSm-*jump*: p<0.01, z = 3.8). The control condition, looking at the blank screen before jumping, did not affect the height of the jump (p = 1.0, z = 0.05). In any case there was no observable significant difference between pronouncing *jump* and reading it fixed or moving bottom-up. The normalization towards BJ gave results comparable to the absolute values (from 106.9±8.9% for RSm*-jump* up to 108.9±11.0 for *jump*; see Table 3 for statistical significance) indicating that reading and pronouncing were similarly effective.

### Experiment 4

The pronunciation of verbs such as *lick* and *pinch* did not show statistically significant effects (Table 2; p = 0.78, z = 1.14 and p = 0.13, z = 2.34, respectively). In semantic coherence, the meaningless verb *tiáo* did not affect the height performance (p = 0.99, z = 0.84) and the normalisation of the values with respect to BJ (Table 3) gave similar results, excepted for *pinch*, which showed a statistically significant improvement in the height of the SVJ (104.4±8.7%, p = 0.043, z = 2.78), when normalized to BJ (Table 3). However, note that hearing *pinch* (H-*pinch*) did not show any significant improvement (see next experiment).

### Experiment 5

The possible effect of hearing (H) these action verbs (Table 2 and 3) gave similar results when compared to their pronunciation. H-*jump* ameliorated significantly the SVJ height (p<0.001, z = 4.13) but neither did H*-lick* (p = 0.30, z = 1.94) nor H*-pinch* (p = 0.83, z = 1.04). The height of SVJs after hearing the meaningless verb *tiáo* was almost identical to that observed in BJs (p = 0.99, z = 0.21). The comparison of the normalized values between pronounced and heard action verbs gave similar results (Table 3).

### Experiment 6

This experiment (Table 2) showed that the height of the jumps was enhanced by the action verb *jump* (p = 0.006, z = 3.49) and significantly affected by the loud pronunciation of *win*, a verb which may have an emotional association to the physical performance (p<0.001, z = −4.1). However a significant effect was also found with the antonym *lose* (p = 0.018, z = 3.19) and this was confirmed when the values were normalized to BJ (table 3; p = 0.014, z = 3.25). The loud pronunciation of *dream* did not affect the performance of the jump (p = 0.64, z = 1.54) and the same was observed with the non-specific action verb *move* (p = 0.36, z = −1.96). The meaningless verb *tiáo* was not included in this experiment in order not to burden the task of the subjects with too many stimuli and risk an unwanted sense of fatigue in the subjects.

### Experiment 7

The last experiment of the study (Table 2 and 3) was performed to further control the effect of verbs signifying actions opposing the SVJ. To further test the reproducibility of the protocol with other subjects that French nationals, the experiment was performed at the Department of Physiotherapy, Lebanese University (the subjects were students at the Department of Physical Education). The results show a significant group effect (p<0.001, F = 31.86) with the expected increase of about 2 cm after hearing the verb *jump* (31.7±0.4 vs 29.8±0.9 for BJ, p<0.001, z = 7.97) and no effect after *fall* (29.8±0.7, p<0.99, z = - 013) or *stop* (29.9±0.7, p<1, z = 0.1).

### General Comments Concerning the Experimental Setting

It is important to note that all the subjects participating to the study were asked, at the end of each experiment, if they had carefully understood the meaning of the verbs and this was confirmed; moreover the subjects stated that they kept obeying, throughout the experiment, to the instruction given at the beginning of the experiment to execute maximum SVJ (this was necessary to avoid inevitable large variations in the values of the heights).

Furthermore, when all the values obtained with the stimulus *jump*, as delivered in experiments 2 to 7 (Table 2), were summed up the results gave an improvement of 2.0 cm (32.8±4.2 cm vs 30.8±4.2 cm for BJ, p<.001, z = 9.43, n = 114) representing an average increase of 106.8±7.5% of the total jump height (p<.001, z = 8.99). Moreover, when all the values obtained, in each experiment, by the action verb *jump* were compared one to the other no statistically significant difference was observed among them (the group effect gave p = 0.88, F = 0.35 when analysed by a one factor ANOVA). Improvements were also obtained with MS (104.4±7.8%), KI (104.6±5.8%) and with the verbs *pinch* (104.4±8.7%; but not H-*pinch*, 101.2±4.4%), *win* (104.9±5.8%) and *lose* (103.6±5.5%) (see Table 3). The meaningless verb *tiáo* did not produce any significant increase in height and its value was quite similar to that of BJ, much as the observation of a blank screen in experiment 3, and verbs *lick*, *dream*, *move*, *fall* and *stop* showed also no statistically significant effect.

## Discussion

The relationship between language and action is based upon experimental evidence reporting the involvement of pre-central frontal cortical areas (premotor, primary motor and Broca’s areas) in the expression of language ([10], for a review see: [9], [53]) and in action understanding [54]. Conversely, it has also been found that language, and in particular action verbs, may stimulate motion. This was done with kinematic studies, usually after reading action words, and by measuring the velocity in grasping or reaching [11], [12]. The observed results showed functional activations in corresponding cortical motor areas [3], [8]. The present findings confirm the relation between language and motion and show that the pronunciation, the enunciation, the hearing and the reading of a specific action verb (*jump*) improve subjects’ performance of a complex movement as a SVJ. Furthermore, the height improvement produced by this action verb is comparable to the effects produced by KI, which is a well-known stimulus used to enhance physical performance (see for review: [55]).

One of the findings arising from studies reported in the literature shows the possibility that the cerebral cortex, organised in specialised areas, may not appear as clearly structured as it was represented in Talairach and Tornoux’s [56] atlas of the brain cortex (see for example: [40], [41]). It is a common observation among neuro-anatomists and, particularly, neurosurgeons that the brain circumvolutions are as complex and varied as human fingerprints and, consequently, that each brain is hardly similar to another one. In fact, already Penfield and Boldrey, in their seminal article on intra-cortical electrical stimulation in epileptic patients [57], observed that the brain cortical differences are not only anatomo-morphologic but also functional. Other findings have shown that the relations among word listening, pronunciation and comprehension, and the motor areas imply their participation and interaction into peri-Sylvian circuitries that modulate these functions (see: [9], [58], [59]). As a consequence the possibility that the brain functional activity corresponds to the regulation of compartmentalised, highly specialised cortical areas does not seem to be confirmed by recent findings as more complex interactions correlate one area to the other through very intricate circuitries (for review see: [18], [20], [21]).

The present findings have led to the main following results: 1. The four types of stimuli that were given to the naïve subjects: loudly and silently pronouncing, hearing and reading a significant and specific action verb (*jump*; at the second person of the French imperative), ameliorate the height of SVJs; 2. Kinaesthetic Imagery, known to stimulate motor action, improved the performance and so did mental subtraction (MS, *vide infra*); 3. When the SVJ was preceded by the pronunciation of action verbs having no specificity in relation to SVJs (*lick* and *pinch*) a statistically not significant increase in the SVJ height was usually observed. The only exceptions appeared with *pinch* after normalization of the data to BJ (although at the limit of significance, p = 0.043, z = 2.78); 4. Verbs capable of raising an affect but not specific to SVJ, as *gagne* (*win*) and its antonym *perds* (*lose*), increased the height of the SVJ, while a verb having no relation to motor action but a peculiar emotionality (*dream*) led to a not significant effect; similarly, a verb having a relation to movement (*move*) was not effective; 5. The addition of a visual stimulus, i.e.: when the specific action verb *jump* was read on a screen, immobile or moving from bottom up at a steady pace, it led to an improvement of the jump height. The effect of reading *jump* was not different to pronouncing it (without visual stimulus); 6. All verbs used in the study, however, increased, even if not significantly, the SVJ height (with the exception of the senseless *tiáo* and blank screen when reading in experiment 3; see Tables 2 and 3), possibly implying that verbs having a poor specificity and an affective touch, may increase the height, as it was observed in the case of *win* and *lose* (Experiment 6, Tables 2 and 3) (it should be kept in mind though that these two verbs may be related to a physical effort, contrary to *dream* and *move*, which in fact showed no statistically significant influence on the jumps; emotions might then be important in influencing the motor action [60], [61], particularly if related to the motion); 7. In an attempt to assure the specificity of hearing the verb *jump* a seventh experiment was performed to compare its effect to that of *fall* and *stop* (Table 2 and 3). The two verbs opposing to the motor action did not show any effect, much as it was observed with *tiáo*. It might have been expected a decrease in the value of the jumps, but the results are in line with the instruction given at the beginning of each trial, to each subject individually, to execute a maximum SVJ. This confirms that the not significant effects observed in table 3 were possibly the consequence of the *intention* and *attention* of each individual to the given task, i.e.: to perform a maximum SVJ regardless the given stimulus (see below).

An interesting result concerned the effect of simple calculus. Arithmetic is known to activate motor brain areas when an exact answer is demanded [62], [63]. Such correlation between simple calculus and the activation of motor areas was the reason that brought to the use of the mental subtraction in the present study, even if the main purpose was to use it as a control for *jump*; the results showed a significant improvement of the SVJ heights after mental subtraction (Table 2 and 3). A possible explanation for this effect may be the reported activation of language areas while calculating in mother tongue (French in this case) [64] and of motor areas found during exact arithmetic [65]. Moreover, and contrary to *tiáo* (senseless verb for whoever is not a sinophone), arithmetic has a significance for all humans representing, much as movement and gestures, a particular form of communication [66]. If this is the answer for the observed effect of calculus upon SVJ height, it may be understandable that a form of communication might improve motor action, probably because of the primitive information qualities of motor actions (gestures), quite common in several cultures in support to language (see: [23]).

The effects observed after KI and after visual observation (when reading) of the action verb require some further comments. As it has been mentioned in the introduction, KI is the kinaesthetic feeling of the motor action that, in the present experiments, was used as a control for the action verb *jump*. The fact that, under the present experimental conditions, KI might also improve the performance of the SVJ gives a further impact of kinaesthetic imagery upon a complex motion.

The improvement in performance induced by some of the stimuli used in the study did not completely follow the specificity usually described in the literature. One possible reason in the discordance may be related to the nature of the motor action itself. A Squat Vertical Jump is a rigorous well-known movement used in biomechanical studies for its reproducibility and the number of information that may be derived from a congruous analysis of the motor action. Compared to a grasping or reaching movement, in neuro-functional terms, a SVJ is a more complex movement as it includes the individual’s *intention*, *attention*, *learning*, *automatisms*, *perceptions, understanding, conceptualisation*, etc.; in other terms, the SVJ movement involves the functional activation of most part of the central sensory-motor systems, including the partial participation of the peripheral nervous systems ([67], [68] and for review [21], [24]). From this point of view, the data are in line with the finding that a difference exists in learning and executing movements of different complexity (see [44], [45]).

In an attempt to reach a tentative conclusion for the present results it seems possible that the intention of the individual, the attention that she/he gives to the motor act [67] and the emotional status produced by words [60], [61] may lead to an interaction of language areas with motor areas to eventually improve performance. This may be particularly so in the case of a complex movement as SVJ.

### Conclusion

The present results indicate that specific cognitive stimuli, such as the verb *jump* or KI, improved the performance of SVJs when related to the complex motor action. However, non-specific cognitive stimuli (verbs *win, lose, pinch* and MS) might also implement the jump (see Table 3 and for review see: [21]). Emotions, represented by verbs *win* and *lose,* may play an important role as it has been recently suggested [60] (especially during a physical effort) and the attention of the individual has also been suggested in the interaction between language and motor action [67], which may possibly happen through a mental simulation process [24], [69].

The present data indicate an improving effect of the action verb specific for the SVJ, but they do not seem to indicate an exclusive specificity probably because of the complexity of SVJ. It might also be suggested that it is not only the specificity of the verb that is important but also the intentionality raised in the subject when pronouncing or expressing or hearing any meaningful action verb (for the role of the fronto-parietal cortex in the intention, planning and decision making in sensory-motor actions, see: [68]). This may include other cognitive means such as KI and calculus.
